# Solitary fibrous tumor of the Pelvic cavity: A rare entity with review of literature

**DOI:** 10.1016/j.radcr.2024.06.087

**Published:** 2024-07-27

**Authors:** Sudeep KC, Himani Poudyal

**Affiliations:** a Department of Radiodiagnosis and Imaging, Patan Academy of Health Sciences, Lalitpur, Nepal; b Department of Internal Medicine, Dhulikel Hospital, Dhulikhel, Kavre, Nepal

**Keywords:** Immunohistochemistry, Mesenchymal neoplasm, Solitary fibrous tumor

## Abstract

Solitary fibrous tumor (SFT) arising from adult mesenchymal stem cells is an uncommon vascular tumor of pelvic cavity. While initially thought to be confined to the pleura, pericardium, or peritoneum, recent studies have revealed that SFTs can develop in different areas of the body. Typically, SFTs grow slowly and may not present noticeable symptoms. In this 2 case study, we describe the clinical situation of a 46-year-old female patient and 68 years old male patient who complained of persistent pelvic pain and urinary symptoms respectively. Imaging tests revealed solitary fibrous tumor in the pelvic cavity which was confirmed on histopathology, an unusual location for this type of tumor. This case reports focusses on importance of early recognition and treatment in dealing with this rare tumor.

## Introduction

Formerly known as hemangiopericytoma, solitary fibrous tumor (SFT) is an uncommon vascular tumor that typically presents as a slow-growing mass in middle-aged adults. While the majority of extra-pleural SFTs are benign, approximately 10%-15% have the potential to recur or become malignant [1]. This article focuses on the MR imaging characteristics of pelvic SFT, confirmed through histopathology. Treatment typically involves wide local excision surgery, although there is no standardized approach. This case study highlights the challenges in diagnosing and treating SFT, especially when located within the pelvic cavity.

## Case report

A 46-year-old female with persistent lower abdominal pain for 1-2 months was found to have a 16-week size mass in the right lower abdomen during examination. Further investigations showed normal levels of human chorionic gonadotropin (Beta hCG), Lactate dehydrogenase (LDH), alpha-fetoprotein, cancer antigen 125 (Ca-125) and carcinoembryonic antigen (CEA).

**Fig. 1:**
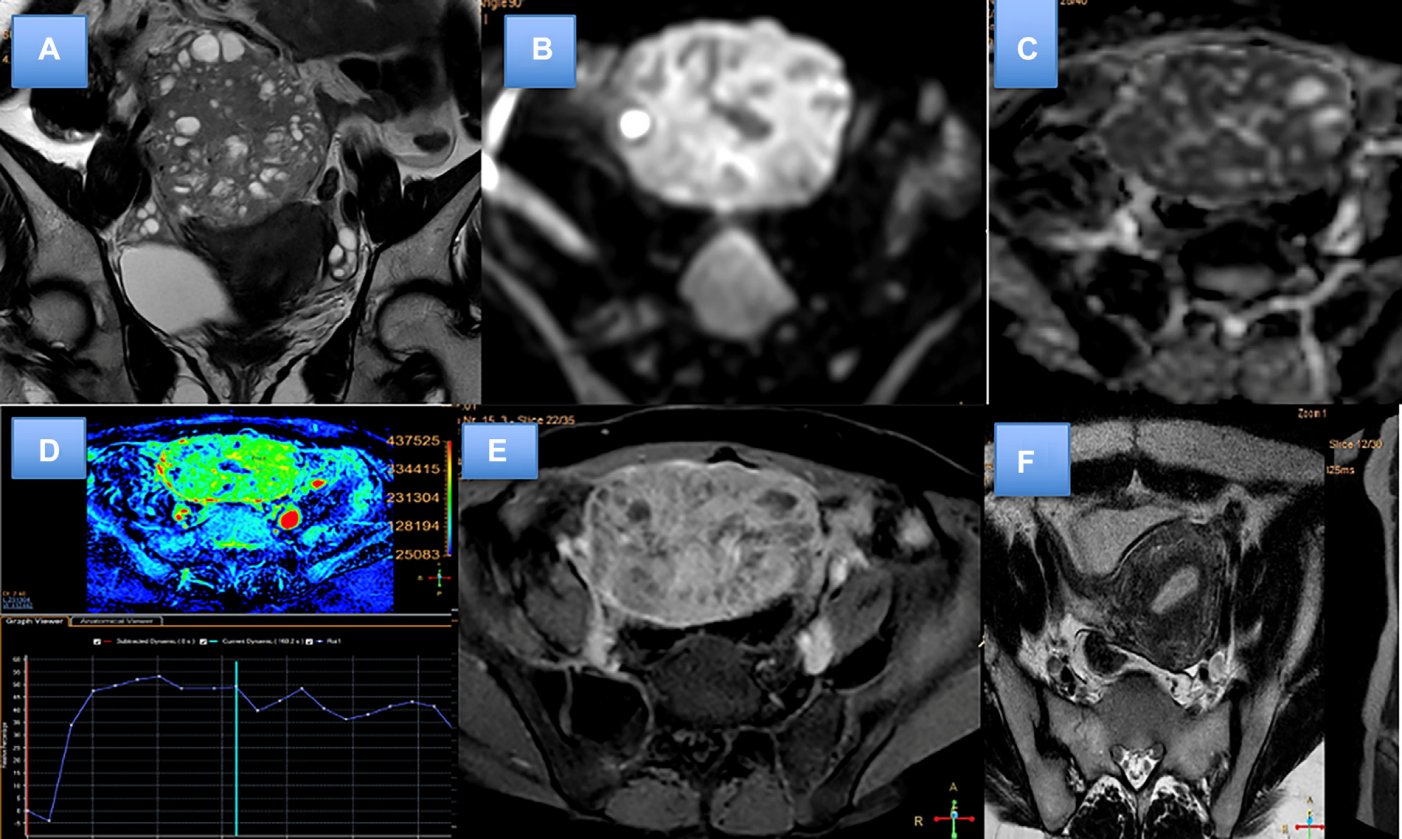
Preoperative imaging findings (A-E). MR images showed a well-defined mass in the pelvic cavity with heterogenous T2 signal intensity and interspersed cystic areas (A). diffusion restriction with high signal on DWI (B) and corresponding low signal on ADC (C). Dynamic contrast study showing type III enhancement curve (D). Heterogenous enhancement seen on post contrast study (E). Postoperative MRI (F) after 6 months showing no recurrence.

Contrast enhanced magnetic resonance imaging (CE MRI) of abdomen and pelvis was done which showed 5.8 × 6.3 × 5.7 cm sized well defined multi-lobulated T1 heterogeneously hypointense lesion with corresponding T2/STIR hyperintensity noted within pelvic cavity separate from pelvic cavity. Multiple T2 hypointense septae/cystic spaces noted within the lesion. Mild diffusion restriction was noted in its soft tissue component with with ADC value of 1.3 × 10^-3mm-2/sec^. Post contrast dynamic study showed avid and progressive enhancement of the lesion and showed delayed washout. No abnormal enhancement or thickening of adjacent peritoneal lining was seen (Fig. 1).

It was considered necessary to perform surgery because of the size of the lesion and the need to rule out the possibility of malignancy. The patient underwent laparotomy and resection. A bilateral salpingectomy was also performed due to the lesion adherence to both fallopian tubes.

Gross examination revealed a distinct lesion covered by a thin translucent peritoneum, showing a grey-white mass with hemorrhagic areas and cystic spaces. Microscopic sections displayed well-defined tumor cells with uniform characteristics. The individual tumor cells were uniform, and indistinct cytoplasmic borders, moderate amount of eosinophilic cytoplasm, spindle shaped vesicular nuclei, smooth nuclear rim and small nucleoli. The mass had a high amount of blood vessels and minimal cell division. Immunohistochemical analysis showed strong positivity for STAT-6 and CD34. The proliferative activity Ki67 was approximately 4%. Risk assessment score was Low risk with Total Score of 2([0{age}+1{mitosis}+1{size}+0{necrosis}]). Ascitic fluid analysis was normal.

The patient's postoperative recovery was unremarkable, with pelvic pain and urinary symptoms resolved. The postoperative period was uneventful, and the patient was stable when discharged. Patient was followed up every 3 months. Follow-up imaging after 6 months showed that the tumor had been completely resected (Fig. 1F). No postoperative adjuvant radio-therapy was given due to low risk. Patient is under regular follow up and shows no sign of recurrence till date (2 year of postsurgery). Patient is planned for 6 months follow up for 5 years and then yearly for next 10 years with ultrasonography of abdomen/pelvis and Chest X-ray as part of follow up.

## Case report 2

**Fig. 2:**
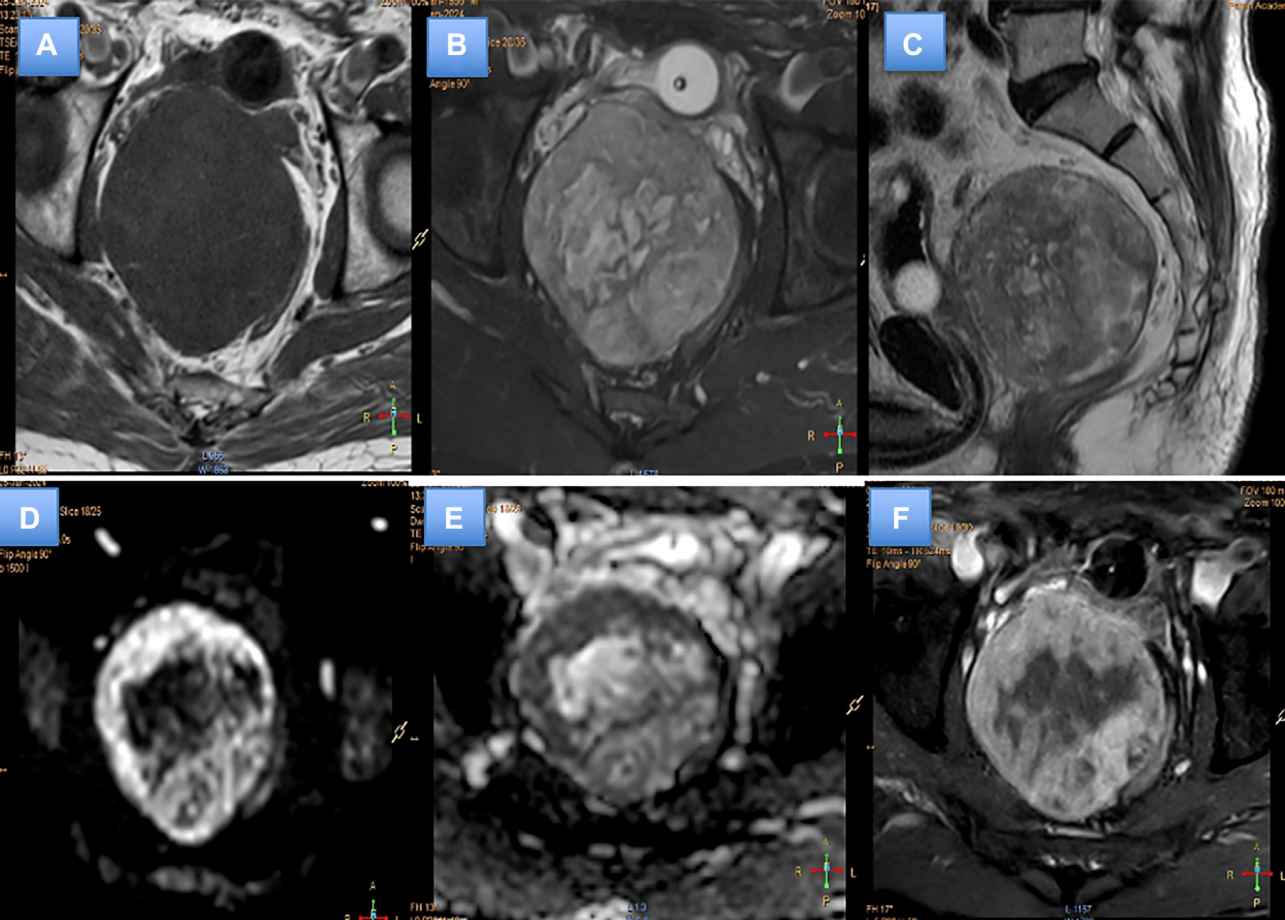
Plain and contrast enhanced MRI of pelvis showing well defined T1 hypointense (A), T2 heterogenous (B) with interspersed T2 hyperintense lesion displacing the urinary bladder and prostate anteriorly. Peripheral diffusion restriction with high signal on periphery (D) and corresponding ADC hypointensity (E) on b-value of 1000. Postcontrast study (F) showing heterogenous enhancement with central nonenhancing areas.

A 68-year-old male patient came to the emergency department with acute urinary retention. He had symptoms of dribbling, incomplete bladder emptying, burning sensation during urination, frequent urination at night. Rest of the medical history was unremarkable. Physical examinations showed normal results, except for an enlarged prostate with a smooth surface and possible involvement of the rectal mucosa. Foley's catheterization was done. An ultrasound revealed a lesion in the pelvic cavity, but the prostate gland was not separately visible. PSA levels were normal. Subsequently, the patient underwent a contrast-enhanced MRI of the abdomen and pelvis which showed heterogenously enhancing well defined circular lesion displaying T2 heterogenous signal intensity and peripheral diffusion restriction as shown in Fig. 2.

Patient underwent transrectal guided (TRUS) biopsy. Microscopic Findings showed linear tissue composed of tumor arranged in hypocellular and hypercellular areas arranged in short fascicles and haphazard pattern. These individual tumor cells were spindle shaped with mild to moderate degree of pleomorphism. Hypocellular areas showed tumor cells surrounded by collagenous stroma. Mitosis constituted 1/10 hpf. Necrosis was also noted comprising approximately 12% of the submitted specimen. Immunohistochemistry showed Strong and diffuse positivity for STAT6 and CD34; CD117 and Desmin was Negative. Mitosis: 1/10 HPF. This tumor was classified as "Intermediate risk” and was planned for surgical excision. Unfortunately, patient expired due to cardiac arrest during surgery.

## Discussion

Solitary fibrous tumors (SFT) are a rare fibroblastic mesenchymal neoplasm characterized by spindle-shaped cells that produce fibrous collagen and mucoid material [1]. The first documented cases of SFT in the pleura were reported by Lietaud in 1767 and Wagner in 1870. In 1991, Witkin and Rosai presented the first case of a head and neck SFT. Originally classified as hemangiopericytomas (HPCs), the World Health Organization has since updated the terminology in 2016, reserving the term solitary fibrous tumor (SFTs)-HPC entities on a single classification system [2].

Solitary fibrous tumors (SFT) are mainly found in the pleura, accounting for less than 5% of pleural tumors. However, they can also occur in other parts of the body, with 20% in the abdomen, 10% in the trunk, 8% in the extremities, and 5% in the neck. Extra pleural SFTs are more common than pleural SFTs [3]. Wignall et al. studied 34 cases of SFTs, mostly located outside the thoracic cavity including soft tissues and muscles (50%) and the pelvis (29%) [4]. Previous case series show that pelvic SFTs do not favor a specific gender and typically appear in the fourth decade of life [5]. Our case report involved both male and female patient in sixth and fourth decade respectively.

Patients can present with wide range of clinical symptoms that are not specific to the location, size, or extent of tumor invasion. Symptoms may include abdominal discomfort, bloating, constipation, difficulty urinating, or increased frequency of urination [6]. In rare cases, the tumor may invade the bladder or ureter, leading to the presence of blood in the urine (hematuria). However, neither of our patients displayed this particular manifestation. Instead, 1 patient reported experiencing vague abdominal pain and another patient reported urinary complaints, suggesting that the pain could be attributed to the pressure exerted by the tumor. There was no evidence of the tumor spreading to other structures, indicating that the symptoms were unlikely to be a result of direct invasion.

Rarely, these tumors can cause paraneoplastic hypoglycemia, known as Doege Potter syndrome characterized by hypoglycemia due to the tumor's production of insulin-like growth factor-1 (IGF-1). Additional symptoms may include joint pain, bone and joint abnormalities, and finger clubbing which usually resolve after surgical removal of the tumor [7]. None of these findings were seen in both of our cases.

The diagnostic value of sonographic and radiographic features for solitary fibrous tumors (SFTs) is limited. SFTs are typically observed as hypoechoic masses on ultrasound, but they can sometimes display heterogeneity caused by necrotic and degenerative changes. Despite being vascular in nature, these tumors may not consistently show blood flow on Doppler imaging [8].

Diagnosing the disease poses a significant challenge as it requires consideration of both clinical and radiological factors. Contrast enhanced computed tomography (CECT) shows lesion with attenuation similar to muscle. Internal calcifications might be present in some cases. In, magnetic resonance Imaging (MRI) images, SFTs generally exhibit intermediate signal intensity on T1-weighted images and heterogeneous low signal intensity on T2-weighted images [9]. This variability in signal can be attributed to the histopathological features, which are influenced by intra-tumoral cystic changes, necrosis, or hemorrhage. The contrast enhancement observed on CT and MRI scans can vary from mild to substantial, showing intense arterial enhancement in hypervascular areas, and hypo enhancement in regions with myxoid degeneration and necrosis [10]. Currently, Gadolinium-enhanced MRI is considered the most effective imaging modality for confirming the exact location, size, relationship between the tumor and adjacent structures, as well as for post-treatment surveillance [9].

In a research conducted by Pang et al. it was noted that the flow-void sign was commonly seen in SFT cases (80% of cases) [11]. Similarly, Tateishi et al. study identified enhancing portions that histologically matched areas with a high concentration of cells and microvessels [12]. Another investigation by Rosado-de-Christenson et al. found that isodensity on precontrast CT scans is linked to hypercellular areas and capillary networks, while regions with marked enhancement indicate hypervascular areas. Intermediate enhanced areas were associated with areas of low cellularity, and patchy hypodensities were linked to necrotic, myxoid, or cystic changes [13].

Dynamic contrast-enhanced MRI (DE-MRI) is also useful for assessing SFTs, providing information on blood flow, permeability, and biological behavior. According to dynamic contrast- enhanced MR imaging proposed by Hisatomi et al, the type III (washout pattern) pattern was seen in our case which was similar to study done by Yang et al. [14,15] DWI is useful for evaluating lesions and distinguishing between malignant and benign tumors by analyzing ADC values. In both of our case, the elevated ADC values indicated a benign nature of the lesion, surpassing the established threshold of 1.0 × 10−^3 mm2/s^. It is worth noting there are limited literature available on the application of DWI in assessing SFTs, highlighting the need for additional research to explore its diagnostic utility [15].

Benign solitary fibrous tumors (SFTs) show low activity in positron emission tomography (PET) scans, while malignant SFTs show increased metabolic rate [16]. Preoperative angiography is crucial for diagnosing SFTs as it helps identify blood supply and allows for targeted embolization of feeder vessel to reduce tumor size prior to surgical removal and minimize bleeding during the operation. Role of pre operative biopsy is controversial due to highly vascular nature of the lesion [17].

Histopathologically, SFTs shows spindle cell arranged in "pattern less pattern", abundant blood vessels, varying connective tissue thickness, high cellularity, and diffuse CD34 positivity. CD34, vimentin, CD99, and bcl-2 serve as markers for SFTs, while cytokeratin's, actins, desmin, and S-100 protein are typically negative. STAT6 is now acknowledged as a reliable nuclear marker with diagnostic and prognostic significance for SFTs which was positive in both of our cases [18].

Malignancy is indicated by necrosis, hemorrhagic areas, pleomorphism, and a high mitotic count. In 2017, Demicco et al. proposed a risk stratification model that includes age, mitoses, tumor size, and necrosis [19]. The World Health Organization categorizes SFT as malignant if tumor size >5 cm, high cellularity, nuclear pleomorphism, high mitotic rate, and necrosis, indicating a higher risk of recurrence. SFTs with a high-grade sarcomatous component are also classified as malignant. However, predicting clinical behavior solely based on histology and imaging is challenging, as SFTs can recur or metastasize even without atypical features [20]. Metastasis of malignant SFTs is via hematogenous pathway.

Differentiating pelvic malignancy from other conditions is crucial due to differences in prognosis, outcomes, and treatment approaches. Common differential diagnoses for pelvic SFT include various mesenchymal tissue-originating tumors such as Angiomyolipoma, fibroma, fibrosarcoma, Liposarcoma, leiomyoma, leiomyosarcoma, desmoid, hemangioma, angiosarcoma, and gastrointestinal stromal tumors (GIST). GIST appears as soft tissue masses, arising from the wall of a hollow viscus with an endoluminal or exophytic growth. Hemangioma shows high signal intensity on T2-weighted MR images, with marked “progressive” enhancement pattern on DCE-MRI. Angiosarcoma shows hyperintense signal on T2-weighted images and marked enhancement. Imaging alone is not enough to rule out these differential, and pathology and immunohistochemistry are essential for an accurate SFT diagnosis [21].

En Bloc surgical excision is recommended for SFT to prevent recurrence, with further therapy needed for advanced or relapsing cases [22]. Tumors larger than 10 cm have a higher risk of metastasis. Regular follow-up evaluations are important to monitor for malignancy, relapse, and metastasis, as there is a 20% recurrence rate especially in cases with larger tumor size, positive surgical margins, and tumors exhibiting malignant features. Adjuvant therapies like radiotherapy and chemotherapy are generally not recommended unless the condition is advanced [1].

Tapias et al. suggested specific pathological indicators including tumor origin, morphology, size, cellularity, necrosis, and mitotic activity to monitor patients for tumor recurrence. A score below 3 points indicates a 100% recurrence-free survival period [23]. However, tumor recurrence remains a significant risk factor for mortality in malignant SFT cases, even with en bloc surgery, adjuvant therapy and diligent monitoring [24]. Study by Savu et al. reviewed 34 cases (75.55%) of pleural SFT at the 2-year follow-up and 7 cases (15.55%) at the 5-year follow-up. Among those reviewed after 2 years, 6 had malignant SFTs (75%) and 28 had benign SFTs (62.22%). At the 5-year follow-up, there were 5 cases of benign SFTs and 2 cases of malignant SFTs [25]. Close monitoring is crucial as aggressive forms of the tumor may reoccur after surgery. In a multicentric study by Von Houdt, local recurrence rate after 5 years of surgery was 29%, metastasis rate was 34%. Positive resection margin was linked to local recurrence whereas tumor size >10 cm and high mitosis rate were associated with higher rate of metastasis [26]. Closer follow up for 2 years and long term follow up for 15 years has been suggested for follow up cases of SFT [19].

## Conclusions

Solitary fibroma is rare, with its malignant variant even more uncommon, making early detection challenging. Pelvic SFTs have unique imaging characteristics that aid in preoperative identification. Regular monitoring is important due to the minimal risk of recurrence. Integrating histopathological, immunohistochemical, imaging and clinical evaluations enable a comprehensive approach to assessment and treatment planning, aiming to reduce recurrence rates and enhance treatment outcomes.

## Author contribution

Concept, design, planning: SKC, HP; Literature review: SKC, HP; Case collection: SKC; Draft manuscript: SKC, HP; Revision of draft: SKC, HP; Final manuscript: SKC, HP; Accountability of the work: SKC, HP.

## Data availability

Any required information is available upon request from the corresponding author.

## Disclaimer

The views and opinions expressed in this article are those of the authors and do not necessarily reflect the official policy or position of any affiliated agency of the authors, and the publisher.

## Patient consent

Written informed consent was obtained from the patients for publication along with relevant images. Patients’ identities are not disclosed.
